# Vitamin D and Its Relationship with Obesity and Muscle

**DOI:** 10.1155/2014/841248

**Published:** 2014-08-05

**Authors:** Cristiana Cipriani, Jessica Pepe, Sara Piemonte, Luciano Colangelo, Mirella Cilli, Salvatore Minisola

**Affiliations:** Department of Internal Medicine and Medical Disciplines, “Sapienza” University, Viale del Policlinico 155, 00161 Rome, Italy

## Abstract

The skin synthesis of vitamin D represents the first step of a metabolic pathway whose features have been extensively studied and clarified in the last decades. In particular, the production of active and inactive forms of the hormone and the actions of the corresponding enzymes have offered new insights into the knowledge of vitamin D metabolism. Additionally, the description of the different organs and tissues expressing the vitamin D receptor and its possible functions, as well as its genetic determinants, have allowed focusing on the interrelationship between vitamin D and many physiological and pathological functions. In this context, many studies reported the association between vitamin D and adipose tissue metabolism, as well as the possible role of the hormone in obesity, weight, and fat mass distribution. Finally, many reports focused on the vitamin D-related effects on skeletal muscle, particularly on the mechanisms by which vitamin D could directly affect muscle mass and strength. This paper is mainly aimed to review vitamin D metabolism and its relationship with obesity and skeletal muscle function.

## 1. Metabolism of Vitamin D

It is an old knowledge that skin exposure to sunlight is the main source of vitamin D production [1, 2]; in fact more than 80% of systemic vitamin D_3_ derives from epidermis and the other 20% is obtained through the diet from animal, cholecalciferol (D_3_), or plant, ergocalciferol (D_2_), and through drug supplementations [3].

**Figure 1:**
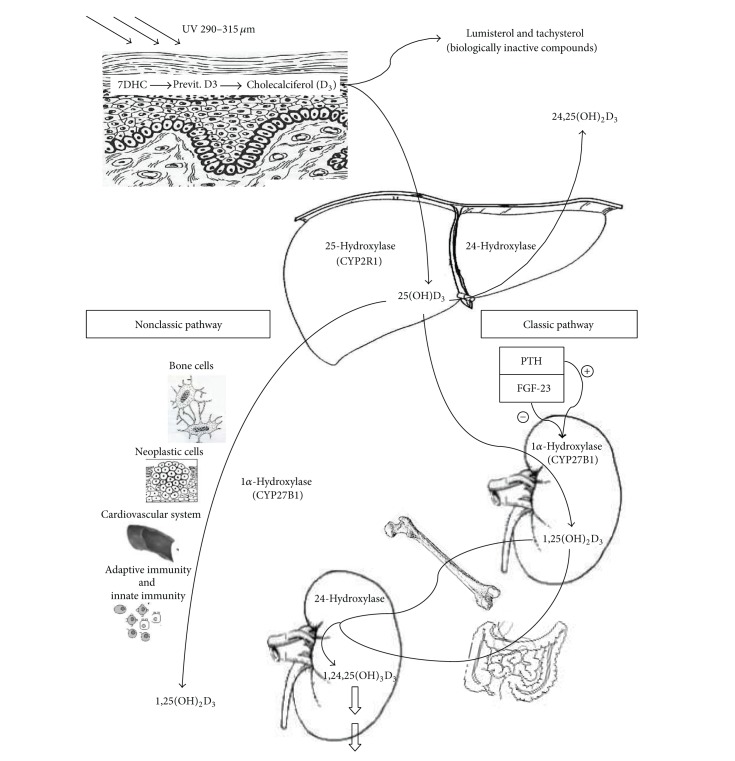
Overview of vitamin D metabolism.

Vitamin D_3_ skin production depends on a photochemical process in which epidermal 7-dehydrocholesterol (7DHC or provitamin D3) is converted to previtamin-D3 (pre-D3) by ultraviolet radiation (UVR) [4] (Figure 1). The so formed pre-D_3_ isomerizes to D_3_ in a thermosensitive but noncatalytic process [5]. To prime sunlight reaction this biochemical process requires specific UVB wavelengths, between 290 and 315 nm, present only for limited number of hours also varying with respect to latitude and season. Therefore, a number of personal and environmental factors are important to maximize the formation of pre-D_3_, like skin pigmentation, clothes, and sunscreen use [1]. However, prolonged exposure to sunlight does not produce toxic amounts of vitamin D_3_ because of the pre-D_3_ conversion to the biologically inactive compounds called lumisterol and tachysterol [6].

In addition to this classical way of vitamin D production, research over the last decade has revealed that numerous pathways for metabolism of vitamin D exist with the production of at least 40 metabolites whose role is only partially known [3, 7].

According to the classical pathway, to become fully active, vitamin D (referred to as either vitamin D_2_ or vitamin D_3_) must be hydroxylated on carbon 25, forming 25-hydroxy-vitamin D [25(OH)D] in the liver, and then on carbon 1, forming 1,25-dihydroxyvitamin D [1,25(OH)_2_D] in the kidney [8]. 25(OH)D is the major circulating metabolite of vitamin D because it has a half-life of 21–30 days [9], so its serum concentration is the most reliable biochemical index of vitamin repletion. 1,25(OH)_2_D is the most potent physiologically active circulating metabolite produced by humans [3]; it has a half-life of 4–15 h [10, 11] and is responsible for serum calcium and phosphate homeostasis via coordinate effects on the kidney, small intestine, and bone [12]. Indeed, it regulates intestinal calcium and phosphorus absorption [13], calcium mobilization from bone, and renal reabsorption of calcium and phosphorus [14].

The conversion of 25(OH)D to 1,25(OH)_2_D depends on the action of the cytochrome P450 enzyme (CYP450), 25-hydroxyvitamin D-1*α*-hydroxylase (1*α*-OHase), in the kidney. However, a cytochrome P27B1 enzyme (CYP27B1), 1*α*-hydroxylase, activity has also been demonstrated in bone cells, both osteoblasts and osteocytes [15, 16]; it leads to a local production of 1,25(OH)_2_D within the osteocytes and directly affects autocrine activities promoting osteoblast and osteocyte maturation and bone remodelling [16, 17]. In recent years 1*α*-hydroxylase activity has been found in other tissues, such as placenta, skin, immune system, and granuloma tissue [18]. Synthesis of 1,25(OH)_2_D in the kidney is directly stimulated by PTH integrating the role of vitamin D in maintaining mineral homeostasis. In fact hypocalcemia, hyperphosphatemia, or reduction in serum fibroblast growth factor 23 (FGF23) results in increased production of PTH that stimulates hydroxylation of 25(OH)D [19]. Conversely, when 1,25(OH)_2_D levels increase, FGF-23 inhibits CYP27B1 in the proximal renal tubule [20]. Additionally, 1,25(OH)_2_D is capable of inversely regulating its own levels by inducing the synthesis of 25-hydroxyvitamin D-24-hydroxylase (24-OHase) [21]. This enzyme is located essentially ubiquitously in all kinds of cells including renal and intestinal cells. The enzyme is also a mixed-function oxidase cytochrome P450 molecule and catalyzes the hydroxylation on carbon 24 leading to the production of 1,24,25-hydroxyvitamin D, the first step in the 24 oxidation pathway that leads to the formation of an inactive water soluble metabolite, calcitroic acid, which is excreted in the urine [22]. 24-hydroxylase produces metabolite also from 25(OH)D leading to the production of 24,25-dihydroxyvitamin D [24,25(OH)_2_D]. Showing the intriguing mechanism in vitamin D metabolism, recently we demonstrated that the administration of high doses of vitamin D leads to a rapid conversion of 25(OH)D in both active and inactive [24,25(OH)_2_D] metabolites [23].

The role and the mechanism of action of these metabolites are not well defined [3]; it could be only hypothesized that if a 24,25(OH)_2_D receptor exists, it would be a member of the nuclear hormone receptor family by analogy with the vitamin D receptor (VDR) [8]. In fact, as a fat-soluble secosteroid hormone, 1,25(OH)_2_D carries out its mechanism of action binding an intracellular receptor that is a member of the superfamily of nuclear receptors. VDR forms a heterodimer with the retinoid X receptor acting as a transcription factor that binds to vitamin D response elements in the promoter region of target genes. This interaction with specific DNA sequences results in the activation or repression of transcription processes. In addition, other ligand-recruited complexes appear to act more directly on the transcriptional apparatus, known as steroid receptor activator complex (SRC) [24]. VDR is expressed both in classical target organs of vitamin D involved in mineral homeostasis and in most tissues and cells of the human body explaining the molecular basis of the pleiotropic effect of vitamin D endocrine-system and its* nonclassical actions *[25]. This system regulates cell proliferation and differentiation and has immunomodulatory, anti-inflammatory, and antifibrotic properties. VDR polymorphisms and different vitamin D metabolisms, involving numerous cytochromes and cytokines, are also considered to be implicated in pathogenetic mechanisms involving numerous systems, for example, cardiovascular [26], metabolic [27], neurological [28], immunological [29], and neoplastic [30] tissues.

## 2. Vitamin D and Obesity

A number of studies have shown that obesity, defined as a body mass index (BMI) ≥ 30 kg/m^2^[31, 32], is associated with low serum 25(OH)D levels [33, 34]. A bidirectional genetic study, which limits confounding, has suggested that higher BMI leads to lower 25(OH)D, each unit increase in BMI being associated with 1.15% lower concentration of 25(OH)D, after adjusting for age, sex, laboratory batch, and month of measurement [35].

The basis of low vitamin D concentration in obesity is still under debate and could be the result of several mechanisms. One hypothesis is that the high content of body fat acts as a reservoir for lipid soluble vitamin D and increases its sequestration, thus determining its low bioavailability [36]. It has also been reported that fat content is inversely related to serum 25(OH)D concentration and that this association is stronger than that between 25(OH)D and BMI [35]. In obese subjects, not only fat mass is increased but also lean body mass, as an adaptative response to greater body weight. In animal studies it has been shown that 25(OH)D was stored 33% in fat and 20% in muscle [37], suggesting that muscle could be also another reservoir of vitamin D in humans. Other authors have theorized that obesity is associated with decreased sunlight exposure, limited outdoor activity, or clothing habits that limits cutaneous vitamin D synthesis [38]. Another hypothesis is that the synthesis of 25-hydroxyvitamin D by the liver may occur at a lower rate in obese subjects due to hepatic steatosis [39]. An alternative explanation is that higher leptin and interleukin 6 circulating levels, mostly secreted by adipose tissue, may have inhibitory effects on 25(OH)D synthesis via their receptors [40]. Even though these previously reported hypotheses may have a role in explaining the reasons for the high prevalence of hypovitaminosis D in obesity, a recent study addresses the question by taking into consideration not only BMI but also body size. This study showed that a volumetric dilutional model accounted for essentially all the variability in serum 25(OH)D concentrations attributable to obesity; in fact once serum 25(OH)D concentrations in obese individuals are adjusted for body size, there is no longer a difference between obese and nonobese individuals [41].

A difference that certainly characterizes obese subjects is the higher fat mass and researchers are now focusing on the interplay between fat mass and vitamin D. Adipose tissue is nowadays considered as a major active endocrine organ secreting heterogeneous bioactive factors, the so-called adipokines [42]. Humans have two major anatomically distinct types of adipose tissues, white and brown which are derived from different cell lineages and exert opposite roles on lipid metabolism. The white fat stores energy and the brown fat dissipates it by using lipids as fuel for thermogenesis. Fat cells are extremely plastic, able to rapidly expand in size and number. In obesity, adipocytes become enlarged with increased macrophage infiltration and a switch towards the proinflammatory phenotype. Interestingly, the ability to both recruit and differentiate new adipocytes is impaired in individuals with hypertrophic adipose tissue [43]. Differentiation into adipocytes requires key transcription factors like the nuclear receptor peroxisome proliferator-activated receptor *γ* (PPAR *γ*) and the CCAAT-enhancer-binding proteins [44].

It has been clearly shown that adipose tissue may both regulate and be regulated by vitamin D [45]. The expression of the vitamin D receptor, 25-hydroxyvitamin D 1*α*-hydroxylase (CYP27B1) genes, and 24-hydroxylase enzyme has been shown in human adipocytes [46]. There are some experimental data suggesting that vitamin D could promote greater adiposity, leading to elevated parathyroid hormone, which may promote calcium influx into adipocytes thereby enhancing lipogenesis [47]. Also 1,25-hydroxyvitamin D modulates adipogenesis through vitamin D receptor-dependent inhibition of critical molecular components of adipogenesis such as peroxisome proliferator-activated receptor *γ* [48]. Data on 1,25(OH)_2_D level are controversial in obese subjects; they are reported to be increased or decreased, probably due to the heterogeneity of the technique used in measuring 1,25(OH)_2_D by immunoassay, which is not totally specific and measures other vitamin D metabolites in serum [49, 50].

The complex biochemical interactions between adipose tissue and vitamin D* in vitro* raise the question as to whether hypovitaminosis D, itself, may contribute to obesity or inhibit weight loss* in vivo*. A few studies have shown that vitamin D, with or without calcium, appears not to have a definite effect on weight, but that it may affect fat mass and distribution. This effect was seen when 25(OH)D level was less than 50 nmol/L; it was not observed when 25(OH)D was above this threshold [51–54]. This demonstrated that giving supplemental vitamin D to those who were replete has no additional effect.

An unresolved question is what dose of vitamin D should be used in obese subjects to replete vitamin D stores and how to maintain normal 25(OH)D levels after repletion. The Institute of Medicine (IOM) guidelines suggest that there is no evidence that increases in vitamin D intake beyond the requirements for nonobese persons can affect bone health or other health conditions among obese persons [55], while Endocrine Society guidelines suggest two to three times more vitamin D in obese people for their age group to satisfy their body's vitamin D requirement [56].

These conclusions are supported by a recent randomized study of seven doses of vitamin D_3_ (from 400 IU/d to 4800 IU/d) showing how the response to vitamin D supplementations was dependent on body size. After vitamin D supplementation, all obese women reached adequate levels of serum 25(OH)D, but women with BMI < 25 kg/m^2^ reached much higher levels of 25(OH)D with the same dose, suggesting that “one size does not fit all”: the dose depends on the threshold of vitamin D to be achieved and on body size [57–61].

However, if the goal is to affect the number of comorbid conditions commonly associated with obesity, where it has been speculated that vitamin D insufficiency may play a role, such as type 2 diabetes [62], cardiovascular disease [63], and hypertension [64], it is likely that the dose of vitamin D required to affect these comorbidities may be different from that needed to suppress PTH [57]. It has been suggested that PTH is suppressed at a lower serum 25(OH)D in obese women compared to the entire population [54]. It is possible that there may be a different set-point for the calcium PTH relationship in the obese, as demonstrated in a calcium-citrate clamp that showed an exaggerated PTH response to hypocalcemia as compared to normal subjects [65]. The etiology for the above is unknown, as well as the dose of vitamin D needed to suppress PTH. Likewise, the dose required to affect comorbidities associated with obesity is uncertain. Considering the effect of vitamin D supplementation on glycaemic indices in obese, 1000 UI/d had no effect [66], while 4,000 to 10,000 IU/d had beneficial effect [67, 68]. Considering the effect on hypertension, a high dose of vitamin D_3_ (15,000 IU/d), in obese hypertensive patients, was demonstrated to reduce tissue-renin angiotensin system activity [69]. Regarding cardiovascular disease risk markers in overweight subjects, a vitamin D supplement of 3332 IU/d was able to significantly reduce triglyceride levels and proinflammatory cytokines [70]. However, Jorde et al. demonstrated that a dose of vitamin D 40 000 IU per week had no positive effect on glucose tolerance, blood pressure, or serum lipids in a sample of subjects with sufficient vitamin D baseline levels [71]. These studies emphasize that only patients with an insufficient vitamin D level would benefit from vitamin D supplements, with a dosage that would appear to be higher than the dose needed to obtain only vitamin D sufficiency and thus PTH suppression. However the mechanisms to explain these results are still largely unknown.

This consideration should be extended also to obese patients who undergo bariatric surgery, which is used with an increasing frequency for weight reduction. Indeed, bariatric surgical procedures may induce malabsorption; therefore, the combination of both low preoperative vitamin concentration and malabsorption may render these patients more prone to severe vitamin D deficiencies. Supplementation with vitamin D should be considered before and after surgery [71]. In any case, clinical studies to determine optimal treatment guidelines for the surgical and nonsurgical population with obesity are warranted.

## 3. Vitamin D and Skeletal Muscle

Vitamin D depletion has been frequently associated with worse physical performance, increased risk of falls, and impaired muscle strength, particularly in the elderly [72–81]. While muscle weakness and pain represent the typical pattern of osteomalacic-associated muscle disease, even atypical clinical presentations are frequent. They include hypotonia, waddling gait, impaired physical function, and uniform generalized muscle wasting and bone pain [82].

Vitamin D exerts an important role in the regulation of skeletal muscle tropism and contraction. As for bone, it has been proposed that vitamin D acts on muscle tissue through both a direct and an indirect effect. The proposed mechanisms include proximal muscle atrophy, loss of type II muscle fibers, and secondary hyperparathyroidism [83–87]. Indeed, vitamin D acts to maintain the function of type II muscle fibers [83, 84]. The histopathological findings showed atrophy of type II skeletal muscle fibers in adults with vitamin D deficiency [85]. This finding is of utmost importance because type II muscle fibers are the first to be recruited when preventing a fall [86].

As far as secondary hyperparathyroidism is concerned, it has been shown that parathyroid hormone negatively affects skeletal muscle function in animal models through proteolysis of muscle proteins and by reducing inorganic phosphate, creatine phosphate, and Ca-ATPase in muscle cells [82].

The direct effects of vitamin D on muscle have to be connected with VDR. Since it was identified in skeletal muscle cells, several reports stated that vitamin D affects muscle function through the binding of 1,25(OH)_2_D to its receptor, resulting in muscle growth, as well as other adaptations [74]. Hence, the role of vitamin D on muscle seems to be connected to the induction of genomic effects, leading to the synthesis of new proteins affecting muscle cell contractility, proliferation, and differentiation and to the regulation of calcium transport in the sarcoplasmic reticulum [87, 88]. Nevertheless, the underlying mechanism is actually not well understood. Data from literature demonstrated that, during development, 1,25(OH)_2_D decreases cell proliferation and enhances myogenic cell differentiation in the mesodermal stem cells by modulating the expression of key pro- and antimyogenic factors, such as IGF-I, IGF-II, follistatin, and myostatin [88]. Hence, 1,25(OH)_2_D can affect myogenic differentiation of skeletal muscle cell lines through an upregulation of* IGF-II* and* follistatin* and a downregulation of* IGF-I* and* myostatin* expression [88]. Garcia et al. demonstrated that the addition of 1,25-dihydroxyvitamin D_3_ to skeletal muscle cells enhanced the expression of myogenic markers and transcription factors at different stages of differentiation [88]. Moreover, after 10 days of incubation of the cells with 1,25-dihydroxyvitamin D_3_, muscle fibers turned to be positive for MHC type II, a late myogenic marker, and showed an increase in the mean diameter and in the width, compared to the controls [88].

Recently, the presence of a functional vitamin D system in muscle, including a CYP27B1 bioactivity, has been demonstrated [86, 87]. This system has been described to act by inhibiting muscle cells proliferation and myotube formation and increasing myotubes size, thus suggesting a direct effect of the hormone on muscle [87]. Conversely, data from Wang and DeLuca demonstrated the absence of vitamin D receptor on skeletal muscle suggesting that the effect of vitamin D in muscle function is most likely indirect [89]. These authors also speculated that the muscle impairment of osteomalacia might depend on associated metabolic changes such as hypocalcemia, hypophosphatemia, and elevated PTH levels [89].

A number of clinical studies have reported that a low vitamin D status is associated with loss of handgrip strength and impaired lower extremity function with increased risk of falls [73–80] (Table 1). Moreover, the effect of vitamin D administration on physical performance, falls, and muscle strength has been widely investigated. Short- and long-term studies collectively demonstrate a relationship between vitamin D status and fall prevention and improvement in muscle strength in community-dwelling older individuals receiving a long-term supplementation with calcium and vitamin D [90–92] (Table 1). Nevertheless, data are still conflicting [74, 81, 93–95]. A meta-analysis of eight randomized controlled trials showed that doses of 700 IU to 1000 IU supplemental vitamin D_3_ a day could reduce falls by 19% or by up to 26% in the elderly [96]. This benefit was significant within 2–5 months and beyond 12 months of treatment; in addition it may not depend on additional calcium supplementation [96]. Active forms of vitamin D were not found to be more effective and vitamin D_3_ has been reported as possibly better than vitamin D_2_ in preventing falls [96]. Finally, based on the possible better efficacy of higher doses of vitamin D, the authors pointed out the need for future research exploring such doses [96]. On the contrary, a double-blind, placebo-controlled trial of 2256 community-dwelling women, aged 70 years or older, considered to be at high risk of fracture, concluded that an annual oral administration of high dose cholecalciferol (500,000 IU) resulted in an increased risk of falls and fractures [97]. Nevertheless, these results were observed early after dosing, being the RR of falls in the vitamin D group 1.31 in the first 3 months (95% CI, 1.12–1.54), but only 1.13 (95% CI, 0.99–1.29) during the remaining months of the year [94].

Other authors found no significant effect of vitamin D supplementation on muscle strength [94, 95]. A more recent study, by Knutsen et al., reported the absence of any improvement in muscle strength or power (as assessed by jump, handgrip, or chair-rising test) after sixteen weeks of daily supplementation with 1,000 IU of vitamin D_3_ in a healthy adult population aged 18–50 years with hypovitaminosis D [98]. Such discrepancies could be due to the lack of homogeneity among the populations studied and the different doses of vitamin D used [93–95]. Indeed, some works actually focused on deficient and others on nondeficient patients and the dose scheme was not adequate in some instances to significantly increase vitamin D serum levels above the threshold of sufficiency [93–95]. On the other hand, the last point is in turn relatedto the fact that the optimal dose and frequency of vitamin D supplementation to achieve and maintain adequate vitamin D serum levels are still debated.

General muscle strength is often evaluated by handgrip strength and/or thigh muscle strength measured by a dynamometer. Gupta et al. reported enhanced handgrip strength in vitamin D deficient Indians aged 20–40 years treated with 60,000 IU per week for 8 weeks followed by 60,000 IU/month for 4 months of cholecalciferol, combined with calcium [90]. In contrast, Goswami et al. reported no improvement in skeletal muscle strength with such a scheduled supplementation [99]. A recent study from our group represents one of the few ones dealing with the issue of muscle strength and vitamin D supplementation in young chronically D-deficient/insufficient people. We evaluated the effect of a single oral dose of 600,000 IU of cholecalciferol on the handgrip strength in young women with vitamin D deficiency [100]. The results showed rapidly improved vitamin D status, while we did not observe any changes in muscle strength parameters in the whole cohort over 3 months, or in a subgroup of women followed up for 6 months. Moreover, 25(OH)D and PTH did not correlate with the two parameters of muscle strength studied at any time point. Finally, we found an increase of serum phosphate in response to vitamin D administration, which could be the most important mechanism of vitamin D effect on muscle, as also suggested by the significant correlation between serum phosphorus levels and muscle strength we found after supplementation both in the whole sample and in the subgroup of women followed up for 6 months [100]. However, the small sample size did not allow concluding the possible mechanisms underlying our results, particularly those related to the effect of high 1,25(OH)_2_ levels on muscle tissue [100].

As discussed in experimental data, clinical studies reported conflicting results, demonstrating that the effect of vitamin D on muscle strength and performance still presents many controversial issues and open questions [101, 102] that need to be addressed also in relation to the reported variation in vitamin D receptor gene [103]. The discordant results are substantially connected to the high variability in terms of study design and muscle parameters considered as outcomes and also reflect the discordant findings on the mechanisms underlying vitamin D and muscle function. Among all, the possible direct effect of the hormone on muscle tissue is still controversial, since opposite data are available on VDR expression on skeletal muscle [83, 89]. Moreover, the metabolic changes associated with vitamin D deficiency have been suggested to be related to a muscle strength improvement after vitamin D supplementation [100]. Hence, given the important action of vitamin D on skeletal muscle tissue, a better understanding of the mechanisms involved is needed, as it will give a new insight into the clinical management of deficient patients.

## 4. Conclusion

Vitamin D represents one of the most studied and discussed topics in the field of bone and mineral metabolism diseases worldwide. The metabolism of the hormone has been extensively clarified, particularly the role of the different enzymes involved, as well as the active and inactive metabolites and the vitamin D receptor. Taken together, these data have also allowed best investigating the pleiotropic and multiorgan-targeted effects of vitamin D. In particular, several studies described the interrelationship between the hormone and the adipose tissue, both considering obesity as a predisposing condition to hypovitaminosis D and vitamin D as a cofactor in the pathogenesis of obesity. Moreover, direct and indirect effects of the hormone on the skeletal muscle tissue lead to a better understanding of the clinical features associated with vitamin D deficiency.

As many efforts have been made in the understanding of vitamin D metabolism and functions, several mechanisms still need to be covered, particularly in relation to many genetic factors involved. Additionally, notwithstanding the whole amount of data on the field, no consensus currently exists on definition and treatment regimen of hypovitaminosis D, mostly as far as particular conditions (such as obesity) and targeting functions (as muscle strength) are concerned.

## Figures and Tables

**Table 1:** Effect of vitamin D on muscle strength and falls.

Author, year, and study type	Patients, age	Endpoints/tools	Result
Visser et al., 2003 [73]; prospective observational study	1008 for grip strength evaluation; 331 for muscle mass evaluation; 55–85 yrs	Grip strength; appendicular skeletal muscle mass (using dual-energy X-ray absorptiometry)	(i) Persons with baseline 25-OHD levels <25 nmol/liter were 2.57 (based on grip strength) and 2.14 (based on muscle mass) times more likely to experience sarcopenia, compared with those with levels >50 nmol/liter (ii) PTH >4.0 pmol/liter was associated with an increased risk of sarcopenia

Latham et al., 2003 [101]; multicenter, RCT∗	243 hospitalized patients; 65 yrs or older	Falls, physical performance (isometric knee extensor strength), and self-rated function	No effect of vitamin D (calciferol, 300,000 IU) on physical health, falls, and physical performance, even in patients with baseline vitamin D levels <12 ng/mL

Kenny et al., 2003 [95]; RCT∗	65 healthy, community-dwelling men; 65–87 yrs	Upper and lower extremity muscle strength and power (using a leg press and handgrip strength), physical performance (specific tests), and activity (using questionnaires)	(i) Baseline 25OHD correlated with baseline single-leg stance time and physical activity score. Baseline PTH levels correlated with baseline 8-foot walk time and physical activity score (ii) No significant difference in strength, power, and physical performance between groups (cholecalciferol 1,000 IU/d or placebo for 6 months, all received 500 mg of calcium)

Broe et al., 2007 [75]; secondary data analysis of a previous RCT∗	124 nursing-home residents; 68–104 yrs	Falls	Supplementation with 800 IU of cholecalciferol reduced the adjusted-incidence rate ratio of falls by 72%, compared to placebo; no differences for the 200, 400, and 600 IU dose

Bischoff-Ferrari et al., 2004 [78]; population-based survey	Ambulatory population; 60–90 yrs	Lower-extremity function; timed 8-foot walk test; and repeated sit-to-stand test	The group in the highest quintiles of 25(OH)D had an average decrease of 0.27 s in the 8-foot walk test and an average decrease of 0.67 s in the sit-to-stand test

Gerdhem et al., 2005 [77]; prospective observational study	986; 75.0–75.9 yrs	Gait, balance, and self-estimated activity level thigh muscle strength	25OHD correlated with gait speed (*P* < 0.001), balance test (*P* < 0.001), self-estimated activity level (*P* < 0.001), and thigh muscle strength (*P* = 0.02)

Houston et al., 2007 [81]; post hoc analysis of a prospective population-based study	976; 65 yrs or older	Short physical performance battery (SPPB) and handgrip strength	(i) Vitamin D levels were significantly associated with SPPB score in men (*P* = 0.04) and handgrip strength in men (*P* = 0.004) and women (*P* = 0.01) (ii) Men and women with serum 25OHD <25.0 nmol/L had significantly lower SPPB score; and those with serum 25OHD <50 nmol/L had significantly lower handgrip strength than those with serum 25OHD ≥25 and ≥50 nmol/L, respectively, (*P* < 0.05) (iii) PTH was significantly associated with handgrip strength only (*P* = 0.01)

Pfeifer et al., 2009 [91]; double-blind, controlled trial	242 community-dwelling people; 70 yrs or older	Falls, body sway, timed-up-and-go test, and maximum isometric leg extensor strength (assessed with a strain gauge dynamometer)	(i) Calcium plus vitamin D significantly decreased the number of subjects with first falls of 27% at month 12 and 39% at month 20, compared to calcium alone (ii) Significant improvements in quadriceps strength of 8%, a decrease in body sway of 28%, and a decrease in time needed to perform the TUG test of 11%

Moreira-Pfrimer et al., 2009 [92]; prospective, double-blind, placebo-controlled, randomized trial	46 patients in long-stay geriatric care, 62–94 years	Maximum isometric strength of hip flexors (SHF) and knee extensors (SKE), measured by a portable mechanical dynamometer	SHF was increased in the calcium/vitamin D group (1 g calcium + cholecalciferol 150,000 IU once a month for the first 2 months and then 90,000 IU once a month for the last 4 months) by 16.4% (*P* = 0.0001) and SKE by 24.6% (*P* = 0.0007), no improvement in the calcium + placebo group

Kukuljan et al., 2009 [93]; RCT∗	180 healthy men, 50–79 yrs	Total body lean and fat mass (DXA^∧^), midfemur muscle cross-sectional area (quantitative computed tomography), muscle strength, and physical function	Daily consumption of low-fat fortified milk (providing 1000 mg calcium and 800 IU vitamin D_3_, per day) does not enhance the effects of resistance training exercise on skeletal muscle size, strength, or function

Bischoff-Ferrari et al., 2009 [96]; meta-analysis of RCT∗	2426 patients from 8 RCT	Falls	(i) High dose supplemental vitamin D reduced fall risk by 19% (ii) Achieved serum 25 (OH)D concentrations of 60 nmol/L or more resulted in a 23% fall reduction

Lips et al., 2010 [94]; double-blind, placebo-controlled trial	126 patients with vitamin D insufficiency; 70 yrs or older	Mediolateral body sway and short physical performance battery (SPPB)	(i) After 16 wk, mediolateral sway and SPPB did not differ significantly between treatment groups (vitamin D_3_ 8400 IU/week versus placebo) (ii) In the post hoc analysis treatment with vitamin D_3_ significantly reduced sway compared with placebo (*P* = 0.047) in patients with elevated baseline sway

Gupta et al., 2010 [90]; double-blind, randomized trial	40 healthy volunteers; 20–40 yrs	Handgrip and gastrosoleus dynamometry, pinch-grip strength, respiratory pressures, 6-minute walk test, and muscle energy Metabolism on ^31^*P*^^ magnetic resonance spectroscopy	The supplemented group (60,000 IU D_3_/week for 8 weeks followed by 60,000 IU/month for 4 months + 1 g of calcium daily) gained a handgrip strength of 2Æ4 kg; gastrosoleus strength of 3Æ0 Nm; and walking distance of 15Æ9 m over the placebo group

Murad et al., 2011 [76]; meta-analysis	45,782 participants from 26 trials	Falls	Vitamin D use was associated with statistically significant reduction in the risk of falls (odds ratio for suffering at least one fall, 0.86; 95% confidence interval, 0.77–0.96)

Goswami et al., 2012 [99]; RCT∗	173 healthy females, mean age 21.7 + 4.4 yrs	Handgrip and pinch grip strength and distance walked in 6 min	Mean handgrip strength and its increase were comparable in 4 groups (double placebo, calcium/placebo, cholecalciferol/placebo, and cholecalciferol/calcium at 6 months)

Cipriani et al., 2013 [100]; prospective intervention study	18 women with vitamin D deficiency (25–39 yrs)	Handgrip strength (using a dynamometer and evaluating maximal voluntary contraction (MVC) and speed of contraction (*S*))	(i) No significant change in MVC and *S* values after vitamin D supplementation (cholecalciferol 600,000 IU) (ii) A significant correlation between MVC and *S* and serum phosphorus after supplementation (*P* < 0.02 and *P* < 0.05, resp.)

Knutsen et al., 2014 [98]; RCT∗	251 healthy adults with vitamin D deficiency (18–50 yrs)	Jump height, handgrip strength, and chair-rising test	(i) Percentage change in jump height did not differ between the group receiving vitamin D_3_ (1000 IU daily) and placebo (*P* = 0.44) (ii) No significant effect of vitamin D on handgrip strength or the chair-rising test

*Randomized controlled trial.

^∧^Dual-energy X-ray absorptiometry.
